# International Clinics

**Published:** 1892-04

**Authors:** 


INTERNATIONAL CLINICS.—Edited by John M. Keating, M. D., J. P. Crozer Griffieth, M. D., J. Mitchell Bruce, M. D., F. R. C. P., David W. Finlay, M. D., F. R. C. P. Published by J. B. Lippincott Co., Philadelphia.

Volume III. of this work shows with what careful selection and arduous labor it has been compiled, and altogether fully maintains that degree of excellence which has hitherto characterized those volumes preceding it.

Beginning as it did by way of expeiiment, it has from the first, met with universal favor, and to-day occupies a conspicuous place in the library of nearly every progressive man in the profession.

For those which are to follow, we bespeak a success as unprecedented as that of its predecessors.

